# Shall We Screen Lung Cancer with Volume Computed Tomography in Austria? A Cost-Effectiveness Modelling Study

**DOI:** 10.3390/cancers16152623

**Published:** 2024-07-23

**Authors:** Hilde ten Berge, Dianne Ramaker, Greta Piazza, Xuanqi Pan, Bernd Lamprecht, Arschang Valipour, Helmut Prosch

**Affiliations:** 1 Institute for Diagnostic Accuracy, 9713 GH Groningen, The Netherlands; 2 Unit of Global Health, Faculty of Medical Sciences, University of Groningen, 9713 GZ Groningen, The Netherlands; 3 Department of Pulmonary Medicine, Kepler University Hospital, 4020 Linz, Austria; 4 Medical Faculty, Johannes Kepler University, 4040 Linz, Austria; 5 Karl-Landsteiner-Institute for Lung Research and Pulmonary Oncology, Klinik Floridsdorf, 1210 Vienna, Austria; 6 Department of Biomedical Imaging and Image-Guided Therapy, Medical University of Vienna, Vienna General Hospital, 1090 Vienna, Austria

**Keywords:** lung cancer screening, Austria, low-dose computed tomography, high-risk individuals, NELSON

## Abstract

**Simple Summary:**

Lung cancer screening (LCS) aims to detect lung cancer in an early stage. Currently, the majority of lung cancer cases are detected in a late stage of the disease, while early detection of lung cancer is associated with better survival rates compared to late-stage disease detection. This study aimed to assess the cost-effectiveness of LCS compared to no screening program in Austria. LCS exhibited an incremental cost-effectiveness ratio (ICER) of EUR 24,627 per quality-adjusted life year (QALY), indicating that LCS is cost-effective. Moreover, the study demonstrated that LCS could avert 11,906 premature lung cancer deaths. These findings provide valuable insights crucial for evidence-based decision making regarding the prospective implementation of LCS initiatives in Austria.

**Abstract:**

This study assessed the cost-effectiveness of a lung cancer screening (LCS) program using low-dose computed tomography (LDCT) in Austria. An existing decision tree with an integrated Markov model was used to analyze the cost-effectiveness of LCS versus no screening from a healthcare payer perspective over a lifetime horizon. A simulation was conducted to model annual LCS for an asymptomatic high-risk population cohort aged 50–74 with a smoking history using the Dutch–Belgian Lung Cancer Screening Study (NEderlands-Leuvens Longkanker ScreeningsONderzoek, NELSON) screening outcomes. The principal measure utilized to assess cost-effectiveness was the incremental cost-effectiveness ratio (ICER). Sensitivity and scenario analyses were employed to determine uncertainties surrounding the key model inputs. At an uptake rate of 50%, 300,277 eligible individuals would participate in the LCS program, yielding 56,122 incremental quality-adjusted life years (QALYs) and 84,049 life years gained compared to no screening, with an ICER of EUR 24,627 per QALY gained or EUR 16,444 per life-year saved. Additionally, LCS led to the detection of 25,893 additional early-stage lung cancers and averted 11,906 premature lung cancer deaths. It was estimated that LCS would incur EUR 945 million additional screening costs and EUR 386 million additional treatment costs. These estimates were robust in sensitivity analyses. Implementation of annual LCS with LDCT for a high-risk population, using the NELSON screening outcomes, is cost-effective in Austria, at a threshold of EUR 50,000 per QALY.

## 1. Introduction

Lung cancer is the second most common cancer diagnosed, accounting for 11% of all newly diagnosed cancers, and it presents the predominant cause of cancer-related mortality annually, contributing to 19% of all cancer-related deaths in Austria [1,2]. Despite improvements in lung cancer survival in Austria, the average five-year survival rate of lung cancer patients is 20%, largely because the majority of lung cancers are diagnosed in an advanced stage [2]. The five-year survival rate for stage IV patients with lung cancer is 5–8%, while for patients diagnosed with stage Ia and Ib lung cancer, when the cancer is still localized, the five-year survival rate is above 62% and 53%, respectively [3]. 

Screening with low-dose computed tomography (LDCT) can shift lung cancer detection from late to early stage, causing a reduction of lung-cancer-specific mortality of 20% and 26%, as shown by the National Lung Screening Trial (NLST) [4] and the Dutch–Belgian Lung Cancer Screening Study (NEderlands-Leuvens Longkanker ScreeningsONderzoek, NELSON), respectively [5]. Moreover, smaller randomized trials conducted across Europe reported the effectiveness of LDCT screening for the early diagnosis of lung cancer [6,7,8,9,10]. The selection criteria for these European lung cancer screening (LCS) studies and the NLST are age and smoking status. The NELSON study included individuals aged 50–74 and current or former smokers (those who had quit ≤10 years ago) who had smoked >15 cigarettes a day for >25 years or >10 cigarettes a day for >30 years) [5]. Smoking status is important as cigarette smoking is the most dominant risk factor for developing lung cancer, with more than 75% of lung cancer cases linked to cigarette smoking [11]. 

Despite this clear evidence and the new recommendation of the European Union to explore the feasibility and effectiveness of the implementation of national LCS programs [12], only a handful of European countries formally committed to setting up nationwide organized screening programs [13]. The Austrian Society of Roentgenology and the Austrian Society of Pneumology recommended LCS implementation in Austria, and there is an intention to initiate the implementation of screening pilots to evaluate the practical and socioeconomic aspects of LCS in the country [2].

National implementation of LCS amongst smokers and former smokers poses several challenges, including the potential long-term impact of LDCT screening on future costs and health benefits. The treatment landscape has changed with the availability of immunotherapy treatments prolonging the survival of subgroups of lung cancer patients but is expensive and not curative [14,15]. In contrast, surgery for stage I lung cancer is cheaper and curative, suggesting that implementing a LCS program for high-risk patients could increase access to curative treatments and improve survival rates. Economic evaluations like cost-effectiveness analyses are an important tool for policymakers seeking to align healthcare policy goals with budgetary constraints and are increasingly used in the Austrian context of health policymaking [16]. The mixed evidence on the cost-effectiveness of LCS gives rise to performing a study investigating the cost-effectiveness of the volume-based LDCT screening versus no screening in Austria [17]. 

The objective of this study was to assess the cost-effectiveness of annual LCS with volume-based LDCT, using NELSON screening outcomes, for a high-risk population in Austria. The Austrian Health Technology Assessment (HTA) guidelines and the recommendations published by the Austrian Institute for Health Technology Assessment were used to inform the study characteristics and methods [17,18]. The cost-effectiveness analysis has the potential to provide insights into the impact and feasibility of a national LCS program for policymakers in Austria, as both the costs and health outcomes of a LCS program are essential to inform the evidence-based decision and ensure a successful LCS integration into the Austrian healthcare system.

## 2. Materials and Methods

A model was constructed using Microsoft^®^ Excel^®^ (Version 2406 Build 16.0.17726.20078), which was validated previously to explore the cost-effectiveness of a nationwide LCS program with volume CT [19]. In this study, the model was adapted to reflect the situations in Austria, making use of available local Austrian data. The subsequent sections concentrate on diverse input variables utilized in this analysis, given that comprehensive model specifications and assumptions have been extensively expounded upon. 

### 2.1. Cost-Effectiveness Analysis

The primary health outcomes assessed in the analysis were life years gained (LYs) and incremental quality-adjusted life years (QALYs), reflecting changes in health-related quality of life and life expectancy when implementing LCS compared to no screening. In addition, the main economic outputs consisted of the incremental costs of implementing a LCS program and the incremental cost-effectiveness ratio (ICER) per QALY. Lastly, the net monetary benefit was calculated by multiplying the willingness-to-pay (WTP) by the incremental QALYs minus the incremental costs.

The WTP threshold was determined based on the gross domestic product (GDP) per capita in Austria according to the World Health Organization (WHO), given there is no explicit WTP threshold illustrated in the health technology assessment (HTA) guideline in Austria [17,18]. According to the WHO, it is established that a health intervention is cost-effective if the ICER is below 3 times the GDP per capita, and very cost-effective when it is below 1 time the GDP per capita [20]. Austria’s GDP per capita was EUR 49,524 in 2022 [21]. After consulting with local experts, the study established the WTP threshold at EUR 50,000, based on the WHO recommendations of one time the GDP and local context (information regarding the experts can be found in Table S1). The Austrian HTA guidelines and the recommendations were used to inform the methodology for the study, and therefore the analysis was conducted from the healthcare payer perspective and for a lifetime horizon [17,18].

### 2.2. Model Structure

This model consisted of a decision tree and a state-transition Markov model. The decision tree simulated the identification and diagnosis of lung cancer patients, who would either be detected through participation in the LCS program or through standard clinical care. In the screening arm, the eligible population would either undergo an annual screening with volume CT until a confirmed diagnosis or choose not to participate. Screening participants with negative screen results would enter the sequent screening in the next year. The LCS encompassed 17 screening rounds, determined by referencing the mean age of participants (58 years) and the upper boundary of the age inclusion criteria based on the NELSON study (74 years) [22]. Individuals who did not participate in the screening, alongside those in the no screening arm, would be diagnosed through standard clinical care after the presentation of lung-cancer-related symptoms. Asymptomatic lung cancer patients with undetected pre-clinical disease in the no screening arm are defined as the missed individuals. All lung-cancer-diagnosed individuals would enter the state-transition Markov model. This Markov model, based on the natural history of lung cancer, simulated disease progression, survival, and treatments for lung cancer patients by the stage at diagnosis. Lung cancer patients encountered the likelihood of transitioning to the subsequent health state (either the post-progression or death state) in the model, at the interval of every three months. This three-month cycle was based on the clinical guidelines to reflect the treatment course and routine follow-up schemes for lung cancer patients [23].

### 2.3. Model Inputs

Localized Austrian data were used to populate the model to ensure the relevance and practicality of the findings in the context of the Austrian population. Alternatively, evidence from other countries was utilized in the absence of local data. The subsequent paragraphs provide details on the model inputs that were customized to the Austrian local settings. The main model inputs are presented in Table 1. The screening outcome parameters were obtained from the NELSON study [22,24] (Table S2). The NELSON study was chosen as it is the largest European lung cancer screening trial using LDCT, used a 16 detector multi-slice CT scanner [25], and was performed between 2003 and 2015 [5]. 

#### 2.3.1. Eligible Population

The selection criteria used in the analysis to determine the population eligible for screening were age (50–74 years) and smoking rate, similar to the NELSON trial eligibility criteria [5]. In 2022, Austria had 2,893,011 individuals aged 50–74, and the smoking rate was 20.76%, resulting in 600,555 individuals eligible for screening [26,27,28,29]. For the base-case analysis, an uptake rate of 50% was chosen, as suggested by local experts. The adherence rate was set at 100%, as recommended by a literature review evaluating LCS cost-effectiveness [30].

**Table 1:** The main parameters for the base-case analysis.

Parameters	Base-Case Value	PSA Distribution	Reference
General settings			
Discount rate for costs	5.00%	Fixed	[18]
Discount rate for health outcomes	5.00%	Fixed	[18]
Time horizon	Lifetime *	Fixed	[17]
Screening uptake rate	50.00%	Beta	Assumption
Demography and epidemiology			
Total population	8,978,929	Gamma	[26]
Population aged 50–74 years	32.22%	Beta	[27]
Smoking rate	20.76%	Beta	[29]
Lung cancer incidence aged 50–74 years	0.45%	Gamma	[11,26,27,28,29,31]
Stage distribution (no screening)			
Stage I	16.30%	Dirichlet	[32]
Stage II	7.80%	Dirichlet	[32]
Stage III	27.70%	Dirichlet	[32]
Stage IV	48.20%	Dirichlet	[32]
Costs			
Recruitment costs			
Invitation letter	EUR 3	Gamma	[33,34]
GP consult	EUR 22	Gamma	[33,34]
Screening costs			
CT scan	EUR 280	Gamma	[33,34]
Diagnostic costs			
Diagnostic costs for screening detected patients (per person)	EUR 803	Gamma	[2,32,33,34,35]
Diagnostic costs for clinically presented patients (per person)	EUR 1093	Gamma	[2,32,33,34,35]
Treatment costs			
Stage I			
First line treatments			
First 3 months	EUR 8564	Gamma	[36,37]
First year (excluding the first 3 months)	EUR 1956	Gamma	[36,37]
Second year	EUR 736	Gamma	[33,34,36,37]
Second line treatment (per patient)	EUR 32,085	Gamma	[2,36,37,38,39,40,41,42,43,44,45,46,47,48,49,50,51]
Stage II			
First line treatments			
First 3 months	EUR 8215	Gamma	[36,37]
First year (excluding the first 3 months)	EUR 2588	Gamma	[36,37]
Second year	EUR 736	Gamma	[33,34,36,37]
Second line treatment (per patient)	EUR 32,085	Gamma	[2,36,37,38,39,40,41,42,43,44,45,46,47,48,49,50,51]
Stage III			
First line treatments			
First 3 months	EUR 16,606	Gamma	[2,36,37,38,39,40,41,42,43,44,45,46,47,48,49,50,51]
First year (excluding the first 3 months)	EUR 41,502	Gamma	[2,36,37,38,39,40,41,42,43,44,45,46,47,48,49,50,51]
Second year	EUR 13,598	Gamma	[2,36,37,38,39,40,41,42,43,44,45,46,47,48,49,50,51]
Second line treatment (per patient)	EUR 24,795	Gamma	[2,36,37,38,39,40,41,42,43,44,45,46,47,48,49,50,51]
Stage IV			
First line treatments			
First 3 months	EUR 12,529	Gamma	[2,36,37,38,39,40,41,42,43,44,45,46,47,48,49,50,51]
First year (excluding the first 3 months)	EUR 25,442	Gamma	[2,36,37,38,39,40,41,42,43,44,45,46,47,48,49,50,51]
Second year	EUR 2517	Gamma	[2,36,37,38,39,40,41,42,43,44,45,46,47,48,49,50,51]
Second line treatment (per patient)	EUR 24,795	Gamma	[2,36,37,38,39,40,41,42,43,44,45,46,47,48,49,50,51]
Follow up costs **			
CT scan	EUR 280	Gamma	[33,34]
Lung physician consult	EUR 88	Gamma	[33,34]
End of life costs	EUR 7466	Gamma	[37]
Utilities			
Pre-progression state			
Stage I	0.78	Beta	[52]
Stage II	0.78	Beta	[52]
Stage III	0.69	Beta	[52]
Stage IV	0.69	Beta	[52]
Post-progression state			
Stage I	0.69	Beta	[52]
Stage II	0.69	Beta	[52]
Stage III	0.69	Beta	[52]
Stage IV	0.69	Beta	[52]
Survival			
OS (five-year survival rate)			
Stage I	78.63%	NA.	[53]
Stage II	54.90%	NA.	[53]
Stage III	29.24%	NA.	[53]
Stage IV	5.91%	NA.	[53]
D/PFS (one-year D/PFS rate)			
Stage I	87.80%	NA.	[54]
Stage II	87.80%	NA.	[54,55]
Stage III	41.81%	NA.	[48]
Stage IV	40.13%	NA.	[41,56,57]
Background mortality			
Life expectancy by age	General population	Beta	[58]

PSA, probabilistic sensitivity analysis; OS, overall survival; D/PFS, disease/progression-free survival; NA, not applicable. * The mean age of participants was 58 years. Following them for a lifetime is equivalent to 42 years. ** Lung cancer patients received chest-CT examination and lung physician consult twice per year in the first 2 years after the initial treatments, and the frequency was adjusted to 1 time per year after 2 years, according to the clinical guidelines for lung cancer treatment and surveillance.

#### 2.3.2. Lung Cancer Epidemiology

Lung cancer stage distribution at the time of diagnosis was obtained from the Austrian Lung Cancer Audit and applied to the no screening arm and individuals who did not participate in the LCS program [32]. The lung cancer incidence in the eligible population was calculated based on the estimated new lung cancer cases in Austria derived from the International Agency for Research on Cancer (IARC) [31], as well as the estimation that 80% of lung cancers were caused by smoking [11].

#### 2.3.3. Survival 

Overall survival (OS) was used to estimate the transition probabilities of lung cancer patients transiting from the pre- and post-progression to the death health state in the Markov model. Stage-specific OS data were obtained from the International Association for the Study of Lung Cancer (IASLC) [53]. Missed individuals were presumed to follow survival patterns similar to stage II lung cancer patients. Disease-free survival (DFS) and progression-free survival (PFS) reflected the time to disease progression or death. Therefore, the transition probabilities of patients transiting from the pre-progression to the post-progression health state were calculated by subtracting the OS rates from the D/PFS rates. Disease- and progression-free survival data were obtained from both real-world evidence [54] and multiple clinical trials [41,48,55,56,57] (Tables S3 and S4). The OS and D/PFS data were extrapolated based on the methodology illustrated in the NICE technical support document and Guyot et al. to reflect the lifetime horizon, and the software used was R studio (2022.12.0 + 353) [59,60]. Details of the parametric distribution fit and the corresponding parameter values per survival curve are presented in Table S4 and could be used to recreate the curves. All-cause mortality was incorporated based on the Austrian life table [58].

#### 2.3.4. Utilities

Health state utility values (HSUVs) per lung cancer stage were taken from a systematic review and meta-analysis [52]. HSUVs for stage I-II and III-IV patients were 0.78 and 0.69, respectively, and these values were applied to patients in the pre-progression health state. Stage III-IV HSUVs were applied to all patients transited to the post-progression state as the disease deteriorated. For the individuals without a lung cancer diagnosis, age-specific population utility values were applied, and they were obtained from the self-reported EQ-5D-5L questionnaires (Table S5) [61,62]. 

#### 2.3.5. Expenses

Expenses encompassing participant recruitment, screening, diagnostic procedures, treatments, ongoing aftercare care, and palliative care were considered to evaluate the cost-effectiveness from a healthcare payer perspective. Recruitment costs and screening costs were obtained from the Department of Health Economics (DHE) database from the Medical University of Vienna [33,34]. The recruitment costs included an invitation letter and a GP consultation, which local experts anticipate will be the recruitment method for LCS in Austria. In our analysis, all individuals aged 50–74, the age criteria of the eligible population, received the costs for an invitation letter. The GP consult costs were applied to individuals participating in the LCS program. The screening costs included the cost of a CT scan.

Diagnostic costs encompassed the expenses incurred after receiving a positive scan or after the clinical presentation of lung-cancer-related symptoms in the non-screening cohort and the non-participants. In the case of clinical presentation, additional costs were accounted for general practitioner or pulmonologist consultation and CT scans. The diagnostic procedures and their corresponding utilization values were derived from the Austrian Lung Cancer Audit (ALCA), a pilot study aiming at evaluating clinical and organizational factors involved in lung cancer care across Austria [32]. Nevertheless, the unit costs associated with diagnostic procedures were obtained from the German outpatient reimbursement catalog, Austria’s neighboring country, due to the absence of publicly accessible information on Austrian tariffs [35]. The unit costs and utilization metrics used for computing diagnostic expenses are specified in Table S6.

For early-stage lung cancer (stage I–II), the costs associated with initial treatments, encompassing the first year following diagnoses, were computed using unit costs and utilization for each treatment modality, such as surgery, chemotherapy, and radiotherapy [36,37]. For advanced-stage lung cancer (stage III–IV), the initial treatments extended to encompass immunotherapy and targeted therapies. Costs associated with immunotherapy were derived from reports released by the Austrian HTA Institute [38,39,51], and expenses associated with targeted therapies were derived from the information supplied by the Main Association of Austrian Social Insurance Institutions [44]. The usage and treatment duration for these novel therapeutic regimens were determined based on the protocols and outcomes established in respective clinical trials [40,41,42,43,46,47,48,49,50,63]. In addition, utilization rates for immunotherapy and targeted therapies were estimated by gene mutation prevalence in lung cancer patients in Austria, in combination with the observations in a German University Hospital, to supplement the absence of utilization data within the Austrian context, and these estimates were validated by the local experts [2,37,45]. Specifics regarding treatment costs can be found in Tables S7 and S8.

The aftercare consisted of regular chest CT scans and pulmonologist consultations following the initial treatments. Patients underwent chest CT scans and consultations every 6 months during the initial two-year period post-treatment, which then reduced to an annual frequency thereafter [23]. The unit costs for these services were sourced from the DHE unit costs database at the Medical University of Vienna [33] (Table S9). Estimated expenses for second-line treatments in early-stage lung cancer (stage I-II) were derived from the initial six-month treatment costs of stage III lung cancer patients, presuming their progression to a locally advanced stage. Similarly, for advanced-stage patients (stage III-IV), the estimates were extrapolated from the initial six-month treatment expenses of stage IV lung cancer patients. End-of-life costs were considered in the study and applied universally to all lung cancer deaths [37]. 

All costs were adjusted to the year 2022 using inflation rates [64]. Moreover, purchasing power parities (PPP) were employed to convert the costs to fit the Austrian context when data were originally sourced from a German setting, enabling an accurate translation for costs between counties [65]. Local experts validated and endorsed the relevance and applicability of the acquired costs to the Austrian context.

### 2.4. Sensitivity Analysis 

One-way sensitivity analysis (OSA) was performed by altering the deterministic parameter values by 20%, and results were presented in a tornado diagram, providing insights into the influential parameters for the analysis. In addition, probabilistic sensitivity analysis (PSA) was conducted to assess the robustness and variability of the model outcomes by simultaneously varying multiple input parameters within their specified probability distributions, and results were demonstrated in a cost-effectiveness scatterplot. Furthermore, the cost-effectiveness acceptability curve was drafted based on ICER calculations across a range of WTP thresholds, depicting the probability of LCS being cost-effective at different monetary values. 

### 2.5. Scenario Analysis 

Scenario analyses were conducted to test the robustness of the model’s outcomes and explore the potential impact of varying assumptions and parameters on the results. Specifically, scenario analyses investigated the influence of diverse screening uptake rates, with estimations derived from the existing cancer screening programs established in Austria [66,67]. Additionally, scenario analyses explore reduced CT scan expenses, which were obtained from neighboring countries like Germany and Italy through cost-effectiveness studies on LCS [37,68,69]. These costs were inflated and converted using PPP before their incorporation into the analyses. Moreover, scenarios with different time horizons, discounting rates, and screening rounds were analyzed. All the parameters used in scenario analyses are summarized in Table S10.

## 3. Results

### 3.1. Base-Case Analysis

Within the Austrian population, a total of 300,277 individuals underwent LCS, leading to a notable stage shift towards early-stage lung cancer. Specifically, 25,893 additional early-stage (I-II) lung cancer patients were identified, accompanied by a reduction of 5521 cases in late-stage (III-IV) diagnoses. Therefore, LCS resulted in 11,906 premature lung cancer deaths averted and thereby avoided 23.7% more lung cancer deaths (Table 2). 

Recruitment costs for LCS were estimated to be EUR 15.7 million, and the screening cost amounted to approximately EUR 944.9 million (EUR 55.6 million per screening round) over 17 screening rounds. Additionally, the incremental diagnostic costs and treatment costs were approximately EUR 35.6 million (EUR 2.1 million per screening round) and EUR 386.0 million, respectively. However, the treatment cost for stage IV patients was reduced by EUR 174.1 million when comparing LCS with no screening. In summary, the implementation of annual LCS in Austria resulted in an ICER of EUR 24,627 per QALY, based on the cumulative incremental costs of EUR 1.4 billion, and a total QALYs gained of 56,122, from a healthcare payer perspective (Table 2).

### 3.2. Sensitivity Analyses

**Figure 1:**
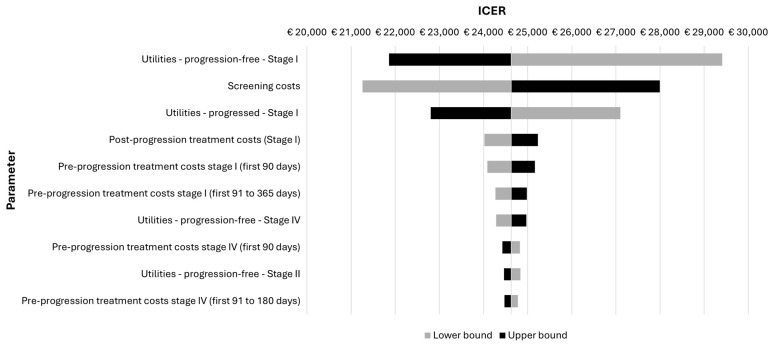
Tornado diagram from the one-way sensitivity analysis.

**Figure 2:**
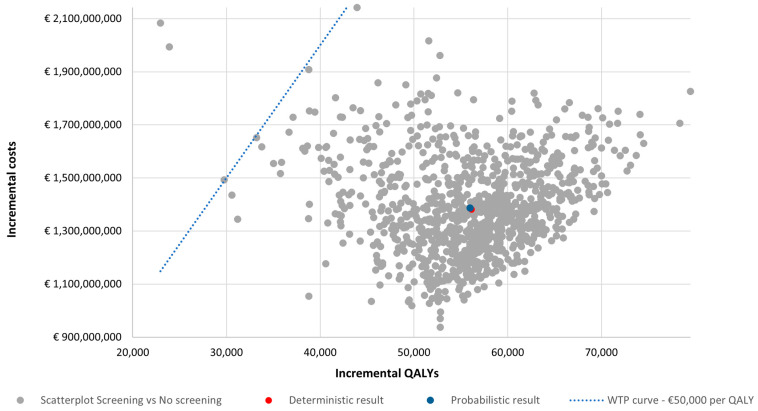
Incremental cost-effectiveness scatterplot from the probabilistic sensitivity analysis. QALYs, quality-adjusted life years; WTP, willingness to pay.

**Figure 3:**
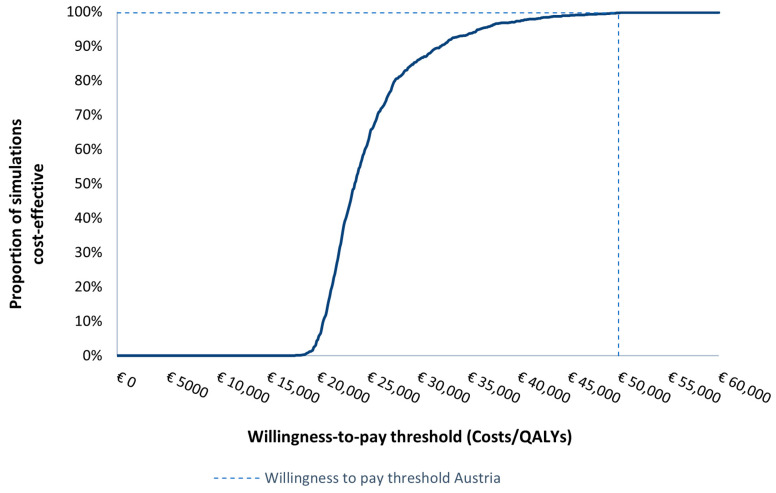
Cost-effectiveness acceptability curve from the probabilistic sensitivity analysis. QALYs, quality-adjusted life years.

One-way sensitivity analysis revealed that the utility values for stage I patients and the CT scan costs were the most impactful parameters affecting the ICER (Figure 1). Nevertheless, OSA resulted in small changes in the ICERs, all within the WTP threshold in Austria. After 1000 iterations, PSA resulted in a probabilistic ICER of EUR 24,777 per QALY (Figure 2) with a cost-effectiveness probability of 99.7% at a WTP of EUR 50,000 (Figure 3). 

### 3.3. Scenario Analysis

Results for scenario analyses are presented in Table 3. The integration of cost reductions associated with CT scans led to a decline in overall screening costs, leading to a decreased ICER for LCS. Moreover, the application of reduced discount rates for both costs and health outcomes (0% and 3%), in contrast to the base-case estimate of 50%, yielded reduced ICER of EUR 14,252 and EUR 20,020, respectively. All scenarios resulted in an ICER well below the commonly used WTP threshold (EUR 50,000 per QALY) in Austria, except for the scenarios with a time horizon of 10 years, indicating an ICER of EUR 109,510 per QALY.

## 4. Discussion

This study investigated the cost-effectiveness of LCS with volume-based low-dose CT versus no screening in Austria, showing an ICER of EUR 24,627 per QALY. These results were robust, as indicated by sensitivity analyses, and probabilistic sensitivity analysis revealed an average probabilistic ICER of EUR 24,777. This is the first modeling study exploring the cost-effectiveness of LCS in Austria, and the findings align consistently with outcomes reported for other cancer screening programs in Austria. Studies showed that colorectal and breast cancer screening in Austria resulted in ICERs of EUR 14,960 and EUR 20,024 per life years gained (LYG), respectively [70,71], which is comparable to EUR 16,444 per LYG found in our study, indicating the reliability of our findings. LCS resulted in a stage shift from late to early-stage detection, with 51% of the lung cancer cases being detected in stage I. This stage shift resulted in 11,906 premature lung cancer deaths being averted. Since the NELSON study, more lung cancer trials have been performed and showed similar or improved outcomes [9,72,73]. In addition, the NELSON study used a 16 row scanner due to technology developments; 64 row scanners or more are replacing 32 row scanners or lower, resulting in higher-resolution images [74]. Therefore, our analysis reflects a conservative approach as improved screening outcome results in a more cost-effective LCS program. 

The one-way sensitivity analysis (OSA) demonstrated that the health state utility values (HSUVs) for stage I lung cancer patients had a substantial impact on the cost-effectiveness of LCS. In our study, HSUVs for lung cancer patients were derived from a comprehensive systemic review, which encompassed aggregated utility values sourced from various countries, thereby constituting a robust and sizable dataset [52]. However, these aggregated values appeared to demonstrate a lower magnitude when contrasted with the average values observed among European respondents [75,76,77,78,79,80], indicating that the utility estimates used in our model might be underestimated, and the quality of life experienced by lung cancer patients in Europe potentially exhibits a higher level when contrasted with that of patients on a global scale. The findings from OSA showed that when the utility value for stage I lung cancer patients increased by 20%, applying 0.936 for progression-free patients and 0.828 progressive patients, LCS would be even more cost-effective, with ICERs of EUR 21,857 and EUR 22,804 per QALY, respectively. 

The cost associated with CT scans was also a pivotal determinant influencing the cost-effectiveness of LCS according to the OSA findings. The CT scan costs used in the model were EUR 280, derived from an online database established by the Medical University of Vienna. However, the CT scan costs used in other CEA studies conducted in neighboring countries seemed to be lower—EUR 69 and EUR 150 were used in German studies, and EUR 80 was used in an Italian study [37,68,69]. Additionally, a systematic review examining the utilization of costing evidence in healthcare decision making reported that there were many uncertainties around the costing data in Austria, primarily attributed to the non-transparent healthcare costing infrastructure [34]. To test the uncertainties surrounding CT scan costs, scenario analyses were conducted, incorporating the above-mentioned reference costs source from Germany and Italy. Findings illustrated a positive correlation between decreased CT scan expenses and heightened cost-effectiveness of LCS, with the ICER ranging from EUR 12,697 to EUR 18,297 per QALY. This implies that LCS would likely demonstrate increased cost-effectiveness within a limited budget allocated for screening expenses if there were reductions in CT scan costs. Research regarding the use of AI as an impartial reader demonstrated that the workload of radiologists can drastically be reduced in the future, which is expected to lower the CT scan costs, resulting in a more cost-effective LCS program [81]. Therefore, efforts should prioritize the strategic management of budget allocations, specifically targeting reductions in CT scan costs in the context of LCS implementation in Austria.

To investigate the effects of LCS uptake rates, scenario analyses were carried out. In the base-case analysis, a 50% uptake rate for LCS was employed. However, relative to the implemented screening programs in Austria, there is potential for the presumed uptake rate of 50% to be optimistically estimated. The breast cancer screening program had an uptake rate of approximately 41% in Austria [82], whereas the counterpart for the colorectal cancer screening program was estimated to be 15.4% to 16.8% [67]. Scenario analyses showed that lowering the LCS uptake rate to 15.4% and 41% in our study would result in ICERs of EUR 24,993 and EUR 24,662 per QALY gained, which demonstrated minimal deviation from the outcomes established in the base-case analysis. This could be elucidated by the observation that the total incremental costs and QALYs exhibited proportional changes across varying screening uptake rates. Nonetheless, increasing the uptake rate yielded more clinical benefits. The additional QALYs gained per patient would escalate from 0.36 to 0.92 with a rise in the screening uptake rate from 15.4% to 50%. Similar trends were observed concerning the screening adherence rate, where the additional QALYs gained per patient was modest at 0.20 when the adherence rate stood at 30%, whereas it substantially increased to 0.92 when the adherence rate reached 100%, though the ICER per QALY showed marginal deviation across different adherence rates; as a reference, the adherence for breast cancer screening was 58% in Austria [67]. Hence, priority should be directed toward enhancing the uptake rate and adherence rate for LCS through strategic interventions and targeted initiatives.

Scenario analyses revealed that the utilization of novel treatments, such as immunotherapy and targeted therapies, had a substantial impact on the cost-effectiveness of LCS. In our study, the treatment utilization data were primarily informed by a study conducted in Germany due to the absence of local data. However, these estimates might be underestimated, as Austria has been reported to be among the countries with one of the shortest durations from the European market authorization until broad access to novel anticancer drugs [83]. Therefore, scenario analyses were conducted to explore an increased utilization of immunotherapy, with a correspondingly decreased usage of chemotherapy and improved survival for late-stage lung cancer patients (stage III and IV) [84]. Results revealed that the enhanced adoption of immunotherapy correlated with a decrease in incremental treatment costs when comparing the screening arm with no screening arm, as the majority of lung cancer patients were diagnosed at an advanced stage in the absence of LCS, and immunotherapy was associated with higher treatment expenses for these patients. Consequently, the ICER per QALY decreased in this scenario, suggesting an enhanced cost-effectiveness of LCS, particularly in the context of widespread utilization of immunotherapy in clinical practice. This indicates that in a prospective scenario where novel anti-cancer drugs are widely accessible, the implementation of LCS would help constrain medical expenses through early detection. Therefore, it holds the potential to impact both the clinical and economic facets of managing lung cancer.

One limitation of the study lay in the absence of comprehensive local diagnostic and treatment cost data, extending beyond the scope of lung cancer evaluation, yet aligning with recognized constraints in health technology assessment methodologies in Austria [34]. A thorough literature review was undertaken to identify and incorporate the most suitable alternative data sources. Additionally, sensitivity analyses were conducted to scrutinize the uncertainties surrounding the costing data, affirming the robustness and reliability of the results. Future research endeavors should aim to offer comprehensive insights into the healthcare costing system in Austria. This could be achieved through the utilization of publicly available databases and conducting micro-costing studies focusing on healthcare expenditures associated with lung cancer, among other potential methodologies.

Secondly, the current analysis focused on the pack-years criteria to select the eligible individuals for LCS. However, current studies, such as the Targeted Lung Health Check (TLHC) program and the 4-in-the-lung-run (4-ITLR) study, focus on optimizing the eligibility criteria for LCS to make screening more personalized [85,86]. Instead of only focusing on the number of pack-years smoked, risk-based screening using lung cancer risk prediction models is investigated. A conservative approach was chosen in the current analysis as it is expected that risk-based screening would enhance the cost-effectiveness of LCS, which was shown in a cost-effectiveness analysis comparing pack-years versus risk-based screening in Switzerland [87]. Moreover, a personalized approach offers the added benefit of potentially reducing radiation exposure by avoiding unnecessary irradiation in lower-risk individuals [74].

Lastly, the UK Lung Cancer Screening (UKLS) trial showed that LDCT screening for high-risk participants is a teachable moment for smoking cessation, especially among those who receive a positive scan result [88]. A prospective study demonstrated that smoking cessation at the time of diagnosis is associated with improved survival for lung-cancer-specific mortality and all-cause mortality [89]. Therefore, the current analysis is a conservative approach as it is likely that more QALYs would be gained when smoking cessation is offered, due to improved survival, which results in a lower ICER.

## 5. Conclusions

Annual LCS with volume-based low-dose CT for a high-risk asymptomatic population, using the NELSON screening outcomes, is cost-effective in Austria, at a threshold of EUR 50,000 per QALY. These findings provide valuable insights crucial for evidence-based decision making regarding the prospective implementation of LCS initiatives in Austria.

## Figures and Tables

**Table 2:** Main results from the base-case analysis for lung cancer screening with volume-based low-dose CT.

	Screening	No Screening	Incremental
Clinical and health outcomes			
Lung cancer diagnoses			
Total	61,039 (100%)	40,667 (100%)	20,372
Stage I	31,182 (51%)	6629 (16%)	24,553
Stage II	4512 (7%)	3172 (8%)	1340
Stage III	12,029 (20%)	11,265 (28%)	764
Stage IV	13,317 (22%)	19,602 (48%)	−6285
Missed individuals	NA.	20,372	NA.
Stage III and IV averted	5521		
Lung cancer deaths			
Total	37,986	49,893	−11,906
Stage I	11,645	2473	9173
Stage II	2394	1681	713
Stage III	10,947	10,244	704
Stage IV	13,000	19,135	−6135
Missed individuals	NA.	16,361	NA.
LYs			
Total	8,143,767	8,059,718	84,049
Stage I	207,142	43,808	163,334
Stage II	23,215	16,070	7145
Stage III	28,653	26,607	2045
Stage IV	13,378	19,668	−6290
Missed individuals	NA.	82,186	−82,186
Lung cancer-free individuals *	7,871,380	7,871,380	0
QALYs			
Total	7,094,500	7,038,378	56,122
Stage I	159,154	33,646	125,507
Stage II	18,071	12,493	5578
Stage III	19,760	18,349	1411
Stage IV	9230	13,570	−4340
Missed individuals	NA.	72,034	NA.
Lung-cancer-free individuals *	6,888,286	6,888,286	0
Cost outcomes			
Total	EUR 2,667,599,196	EUR 1,285,508,127	EUR 1,382,091,070
Recruitment costs	EUR 15,658,995	−	EUR 15,658,995
Screening costs	EUR 944,862,093	−	EUR 944,862,093
Diagnostic costs	EUR 67,205,929	EUR 31,641,587	EUR 35,564,342
Treatment costs	EUR 1,639,872,179	EUR 1,253,866,539	EUR 386,005,640
Stage I	EUR 628,288,448	EUR 133,053,631	EUR 495,234,817
Stage II	EUR 83,909,760	EUR 58,326,545	EUR 25,583,215
Stage III	EUR 557,339,147	EUR 518,006,566	EUR 39,332,581
Stage IV	EUR 370,334,824	EUR 544,479,797	EUR −174,144,972
Health economic outcomes			
ICER (per QALY)	EUR 24,627		
NMB	EUR 1,424,012,194		

LYs, life years; QALYs, quality-adjusted life years; ICER, incremental cost-effectiveness ratio; NMB, net monetary benefit. * Lung-cancer-free individuals refers to the individuals who do not have lung cancer.

**Table 3:** Results from the scenario analysis.

Scenario Name	Screening		No Screening		Incremental Total Costs	Incremental QALYs	ICER
	Total Costs	Total QALYs	Total Costs	Total QALYs			
Base-case analysis	EUR 2,667,599,196	7,094,500	EUR 1,285,508,127	7,038,378	EUR 1,382,091,070	56,122	EUR 24,627
Time horizon—10 year	EUR 1,875,220,197	4,246,046	EUR 892,861,312	4,237,076	EUR 982,358,885	8,970	EUR 109,510
Time horizon—20 year	EUR 2,654,058,718	6,260,895	EUR 1,279,280,611	6,224,315	EUR 1,374,778,107	36,580	EUR 37,583
Time horizon—30 year	EUR 2,666,645,093	6,961,351	EUR 1,285,182,774	6,908,586	EUR 1,381,462,318	52,765	EUR 26,182
Decrease discount rate (costs: 0%, health outcomes: 0%)	EUR 3,817,460,602	12,072,272	EUR 1,852,663,992	11,934,416	EUR 1,964,796,609	137,856	EUR 14,252
Decrease discount rate (costs: 3%, health outcomes: 3%)	EUR 3,049,548,566	8,572,349	EUR 1,473,957,511	8,493,647	EUR 1,575,591,055	78,702	EUR 20,020
Increase discount rate (costs: 10%, health outcomes: 10%)	EUR 2,002,629,443	4,894,393	EUR 957,437,818	4,867,585	EUR 1,045,191,625	26,808	EUR 38,989
Number of screening rounds—3	EUR 755,476,931	7,308,584	EUR 343,798,748	7,290,868	EUR 411,678,182	17,716	EUR 23,237
Number of screening rounds—5	EUR 1,154,543,454	7,262,156	EUR 539,349,004	7,234,965	EUR 615,194,450	27,191	EUR 22,625
Number of screening rounds—10	EUR 1,946,938,252	7,165,845	EUR 929,028,991	7,122,063	EUR 1,017,909,262	43,782	EUR 23,249
Number of screening rounds—15	EUR 2,500,355,235	7,109,451	EUR 1,202,568,477	7,055,872	EUR 1,297,786,758	53,579	EUR 24,222
Screening uptake rate—15.4%	EUR 1,731,160,173	7,108,068	EUR 1,299,135,231	7,090,783	EUR 432,024,942	17,286	EUR 24,993
Screening uptake rate—16.8%	EUR 1,769,050,770	7,107,519	EUR 1,298,583,845	7,088,662	EUR 470,466,924	18,857	EUR 24,949
Screening uptake rate—41.0%	EUR 2,424,016,791	7,098,029	EUR 1,289,052,749	7,052,009	EUR 1,134,964,042	46,020	EUR 24,662
Screening adherence rate—30%	EUR 1,530,137,034	7,107,153	EUR 1,300,473,717	7,098,500	EUR 229,663,317	8,652	EUR 26,544
Screening adherence rate—50%	EUR 1,591,597,866	7,106,196	EUR 1,299,291,176	7,094,502	EUR 292,306,691	11,694	EUR 24,997
Screening adherence rate—70%	EUR 1,719,499,677	7,104,353	EUR 1,297,035,858	7,086,641	EUR 422,463,819	17,713	EUR 23,851
Smoking rate—15%	EUR 1,930,107,627	5,126,375	EUR 928,888,150	5,085,822	EUR 1,001,219,478	40,553	EUR 24,689
Smoking rate—10%	EUR 1,289,792,798	3,417,583	EUR 619,258,767	3,390,548	EUR 670,534,032	27,035	EUR 24,802
Smoking rate—5%	EUR 649,477,969	1,708,792	EUR 309,629,383	1,695,274	EUR 339,848,586	13,518	EUR 25,141
CT scan costs of EUR 82	EUR 1,998,099,825	7,094,500	EUR 1,285,508,127	7,038,378	EUR 712,591,699	56,122	EUR 12,697
CT scan costs of EUR 103	EUR 2,071,380,174	7,094,500	EUR 1,285,508,127	7,038,378	EUR 785,872,048	56,122	EUR 14,003
CT scan costs of EUR 175	EUR 2,312,370,826	7,094,500	EUR 1,285,508,127	7,038,378	EUR 1,026,862,699	56,122	EUR 18,297
Smoking cessation program (EUR 300)	EUR 2,757,682,436	7,094,500	EUR 1,285,508,127	7,038,378	EUR 1,472,174,309	56,122	EUR 26,232
Smoking cessation program (EUR 400)	EUR 2,787,710,182	7,094,500	EUR 1,285,508,127	7,038,378	EUR 1,502,202,056	56,122	EUR 26,767
Increase immunotherapy utilization for late-stage lung cancer patients by 10%	EUR 2,719,836,302	7,095,042	EUR 1,345,885,191	7,039,047	EUR 1,373,951,112	55,996	EUR 24,537
Increase immunotherapy utilization for late-stage lung cancer patients by 20%	EUR 2,756,338,111	7,095,042	EUR 1,384,950,157	7,039,047	EUR 1,371,387,954	55,996	EUR 24,491
Increase immunotherapy utilization for late-stage lung cancer patients by 50%	EUR 2,865,843,537	7,095,042	EUR 1,502,145,055	7,039,047	EUR 1,363,698,481	55,996	EUR 24,354
Increase immunotherapy utilization for late-stage lung cancer patients by 100%	EUR 3,048,352,580	7,095,042	EUR 1,697,469,886	7,039,047	EUR 1,350,882,693	55,996	EUR 24,125
Apply disutility to false positives and indeterminate scans—0.015	EUR 2,667,599,196	7,090,329	EUR 1,285,508,127	7,038,378	EUR 1,382,091,070	51,952	EUR 26,603
Apply disutility to false positives and indeterminate scans—0.03	EUR 2,667,599,196	7,086,159	EUR 1,285,508,127	7,038,378	EUR 1,382,091,070	47,781	EUR 28,925
Apply disutility to false positives and indeterminate scans—0.05	EUR 2,667,599,196	7,080,598	EUR 1,285,508,127	7,038,378	EUR 1,382,091,070	42,221	EUR 32,735
Increase utility values for stage I lung cancer patients by 20%	EUR 2,667,599,196	7,106,650	EUR 1,285,508,127	7,043,913	EUR 1,382,091,070	62,738	EUR 22,030
Increase background mortality by 100%	EUR 2,495,998,363	6,191,043	EUR 1,199,101,303	6,149,484	EUR 1,296,897,061	41,559	EUR 31,206

QALYs, quality-adjusted life years; CT, computed tomography.

## Data Availability

All data presented in this study are available in the article and Supplementary Materials. Further inquiries can be directed to the corresponding author upon a reasonable request.

## Supplementary Materials

The following supporting information can be downloaded at https://www.mdpi.com/article/10.3390/cancers16152623/s1. Table S1: Overview of the Austrian local experts. Table S2: Screening outcomes input parameters for the base-case analysis. Table S3: Clinical trials used to synthesize the progression-free survival data for stage IV lung cancer patients. Table S4: Parametric distributions and the corresponding parameters used to extrapolate survival curves. Table S5: Utility norm used for general population in Austria. Table S6: Unit costs and utilization of diagnostic procedures for lung cancer patients. Table S7: Costs and duration of treatments for lung cancer patients. Table S8: Treatment utilization per lung cancer stage. Table S9: Follow-up costs for lung cancer patients. Table S10: Parameters and their corresponding values used for scenario analyses.
