# Supplementary material for: Shall We Screen Lung Cancer with Volume Computed Tomography in Austria? A Cost-Effectiveness Modelling Study
Source: Cancers (Basel). 2024 Jul 23;16(15):2623. doi: 10.3390/cancers16152623 (PMC11310943; doi:10.3390/cancers16152623)
Supplement: Supplementary file 1 [file cancers-16-02623-s001.zip › cancers-3097339-supplementary.pdf]

Supplementary materials

Supplementary Table S1. Overview of the Austrian local experts.

Name	Affiliate
Prof. dr. H. Prosch	Department of Biomedical Imaging and Image-Guided Therapy, Medical University of Vienna, Vienna General Hospital, 1090 Vienna, Austria
Prof. dr. A. Valipour	Karl-Landsteiner-Institute for Lung Research and Pulmonary Oncology, Klinik Floridsdorf, Vienna, Austria
Prof. dr. B. Lamprecht	Department of Pulmonary Medicine, Kepler University Hospital, Linz, Austria; Medical Faculty, Johannes Kepler University, Linz, Austria Unit of Global

Supplementary Table S2. Screening outcomes input parameters for the base-case analysis.

Screening outcomes		Base-case value	PSA distribution	Reference
NELSON round 1				
Regular scan	Negative	79.21%	Dirichlet	[1]
	Indeterminate	19.20%	Dirichlet	[1]
	Positive	1.59%	Dirichlet	[1]
Indeterminate scan	Negative	94.57%	Dirichlet	[1]
	Positive	5.43%	Dirichlet	[1]
True negative		99.93%	Dirichlet	[1]
False negative		0.07%	Dirichlet	[1]
True positive*		38.67%	Dirichlet	[1]
False positive*		61.33%	Dirichlet	[1]
Stage distribution	Stage I	64.86%	Dirichlet	[2]
	Stage II	9.46%	Dirichlet	[2]
	Stage III	18.92%	Dirichlet	[2]
	Stage IV	6.76%	Dirichlet	[2]
NELSON round 2				
Regular scan	Negative	92.17%	Dirichlet	[1]
	Indeterminate	6.58%	Dirichlet	[1]
	Positive	1.25%	Dirichlet	[1]
Indeterminate scan	Negative	91.23%	Dirichlet	[1]
	Positive	8.77%	Dirichlet	[1]
True negative		99.73%	Dirichlet	[1]
False negative		0.27%	Dirichlet	[1]
True positive*		44.35%	Dirichlet	[1]
False positive*		55.65%	Dirichlet	[1]
Stage distribution	Stage I	75.86%	Dirichlet	[2]
	Stage II	6.90%	Dirichlet	[2]
	Stage III	13.79%	Dirichlet	[2]
	Stage IV	3.45%	Dirichlet	[2]

NELSON, Dutch–Belgian Lung Cancer Screening Study; PSA, probabilistic sensitivity analysis.
**True positive refers to the proportion of true positive scans among all positive results, while false positive refers to the proportion of false positive scans among all positive results. Together, these proportions add up to 100%.*

Supplementary Table S3. Clinical trials used to synthesize the progression-free survival data for stage IV lung cancer patients.

Trial	Patient enrolled	Intervention	Trial design	Weight*
KEYNOTE-189 [3]	616 non-actionable mutations NSCLC patients (advanced stage)	Pembrolizumab + chemotherapy	Phase III double-blind RCT	68%
FLAURA [4]	279 EGFR-mutated NSCLC patients (advanced stage)	Osimertinib	Phase III double-blind RCT	17%
IMpower133 [5]	403 SCLC patients (advanced stage)	Atezolizumab + chemotherapy	Phase III double-blind RCT	15%

NSCLC, non-small cell lung cancer; RCT, randomized control trial; SCLC, small cell lung cancer.
** the weights used to synthesize the survival curves from various clinical trials were based on the epidemiology of lung cancer patients and the prevalence of the gene mutation.*

Supplementary Table S4. Survival curve extrapolation is done using parametric distributions and their accompanying parameters.

	Parametric distribution	Base-case value	Reference
Overall survival			
Stage IA1	Log-logistic distributed - kappa	1.8837	[6]
Stage IA1	Log-logistic distributed - theta	183.6936	[6]
Stage IA2	Weibull distributed - gamma	1.3572	[6]
Stage IA2	Weibull distributed - lambda	192.9953	[6]
Stage IA3	Weibull distributed - gamma	1.1139	[6]
Stage IA3	Weibull distributed - lambda	186.0931	[6]
Stage IB	Weibull distributed - gamma	1.1608	[6]
Stage IB	Weibull distributed - lambda	132.9876	[6]
Stage IIA	Weibull distributed - gamma	0.9189	[6]
Stage IIA	Weibull distributed - lambda	132.5941	[6]
Stage IIB	Weibull distributed - gamma	0.9470	[6]
Stage IIB	Weibull distributed - lambda	95.0181	[6]
Stage IIIA	Weibull distributed - gamma	0.8131	[6]
Stage IIIA	Weibull distributed - lambda	58.7308	[6]
Stage IIIB	Weibull distributed - gamma	0.8077	[6]
Stage IIIB	Weibull distributed - lambda	38.5747	[6]
Stage IIIC	Log-logistic distributed - kappa	1.3979	[6]
Stage IIIC	Log-logistic distributed - theta	12.7206	[6]
Stage IVA	Weibull distributed - gamma	0.8206	[6]
Stage IVA	Weibull distributed - lambda	19.9146	[6]
Stage IVB	Weibull distributed - gamma	0.9168	[6]
Stage IVB	Weibull distributed - lambda	10.0178	[6]
Disease/progression-free survival			
Stage I - Group A*	Log-logistic distributed - kappa	1.3840	[7]
Stage I - Group A	Log-logistic distributed - theta	48.0175	[7]
Stage I - Group B*	Log-logistic distributed - kappa	1.6265	[7]

Stage I - Group B	Log-logistic distributed - theta	60.0375	[7]
Stage II - Group A*	Log-logistic distributed - kappa	1.3840	[7]
Stage II - Group A	Log-logistic distributed - theta	48.0175	[7]
Stage II - Group B*	Log-logistic distributed - kappa	1.6265	[7]
Stage II - Group B	Log-logistic distributed - theta	60.0375	[7]
Stage II - Group C*	Log-normal distributed - mu	3.5337	[8]
Stage II - Group C	Log-normal distributed - delta	1.4040	[8]
Stage III - Group D+	Log-normal distributed - mu	2.6934	[9]
Stage III - Group D	Log-normal distributed - delta	1.4972	[9]
Stage III - Group E+	Log-normal distributed - mu	1.9114	[9]
Stage III - Group E	Log-normal distributed - delta	1.2816	[9]
Stage IV - non-EGFR mutated NSCLC	Log-logistic distributed - kappa	1.5881	[3]
Stage IV - non-EGFR mutated NSCLC	Log-logistic distributed - theta	8.5885	[3]
Stage IV - EGFR mutated NSCLC	Log-normal distributed - mu	2.8941	[4]
Stage IV - EGFR mutated NSCLC	Log-normal distributed - delta	0.9089	[4]
Stage IV - SCLC	Log-logistic distributed - kappa	2.5367	[5]
Stage IV - SCLC	Log-logistic distributed - theta	5.2757	[5]

EGFR, epidermal growth factor receptor; NSCLC, non-small cell lung cancer; SCLC, small cell lung cancer.
* *for lung cancer stage I, a retrospective study was used to inform the disease-free survival, which included two groups of patients, presented as group A and B in the table, and the survival data used were weighted based on the number of patients enrolled in both groups. For lung cancer stage II, besides group A and B sourced from the retrospective study, survival data for group C was obtained from the clinical trial IMpower010 (control arm), as the majority of enrolled patients in this trial were stage II patients, thus group A,B, and C jointly constituted a representative sample for stage II lung cancer patients.*
\+ *for lung cancer stage III, survival data from the trial PACIFIC was used (both arms) and presented as group D and E in the table; these survival data were weighted based on the number of patients enrolled in both groups.*

Supplementary Table S5. Utility norm used for general population in Austria.

Age	Utility value	Reference
45-54	0.945	[10]
55-64	0.922	[10]
65-69	0.92	[11]
70-74	0.85	[11]
75-79	0.82	[11]
80+	0.68	[11]

Supplementary Table S6. Unit costs and utilization of diagnostic procedures for lung cancer patients.

Procedure	Utilization	Unit costs (2022)	Utilization reference	Cost reference
**Imaging studies**				
PET-CT	56%	€675	[12]	[13]
Brain-MRI	45%	€126	[12]	[13]
CT scan*	90%	€280	[12]	[14]
**Invasive procedures**				
Bronchoscopy	88%	€136	[12]	[13]

	CT-guided biopsy[+]	5%	€145	[12]	[13]
	Ultrasound-guided lymph node biopsy[+]	3%	€145	[12]	[13]
	Thoracoscopy or mediastinoscopy	4%	€154	[12]	[13]
**Pathological and gene examination**					
	Histological/cytological confirmation	100%	€29	[15]	[13]
	PD-L1 mutation examination	83%	€157	[12]	[13]
	Targeted genes examination	39%	€157	[12,15]	[13]
**Others**					
Interdisciplinary meetings		56%	€24	[12]	[13]
Diagnostic costs for screening detected patients (per person)				€803	
**Patients referral path with clinical presentations [#]**					
	Patients referred by Lung specialist/ Physicians	60%	€54	[12]	[14]
	Patients referred by General practitioner	20%	€22	[12]	[14]
	Self-referrals	7%	-	[12]	NA.
Diagnostic costs for clinically presented patients (per person)				€1,093	

PET-CT, positron emission tomography-computerized tomography; MRI, magnetic resonance imaging.
[*] the CT scan costs are applied to patients with clinical presentation, as screening detected patients have received CT scans in the screening process.
[+] the costs for CT-guided biopsy and Ultrasound-guided lymph node biopsy are assumed to take the average values of the costs for bronchoscopy and thoracoscopy or mediastinoscopy.
[#] the costs incurred through the referral path are accounted as the additional costs patients with clinical presentation.

Supplementary Table S7. Costs and duration of treatments for lung cancer patients.

Intervention/Timeframe		Costs (Austria, 2022)	Reference
**Surgery with or without chemotherapy**			
	First 3 months after diagnosis	€8,680	[16]
	First year excluding the first 3 months (per 3 months)	€1,746	[16]
	After 1 year since diagnosis (per 3 months)	€3,480	[16]
**Chemotherapy with or without radiotherapy**			
	First 3 months after diagnosis	€6,357	[16]
	Remaining cycles year 1 (per 3 months)	€5,958	[16]
	Remaining cycles year 2> (per 3 months)	€6,057	[16]
**Immunotherapy + (immunochemotherapy)**			
Firstline treatment duration is based on clinical trials [17–21]			
	Pembrolizumab (per 3 months)	€31,045	[22]
	Atezolizumab (per 3 months)	€20,847	[23]
	Durvalumab (per 3 months)	€37,056	[24]
**TKIs**			
Firstline treatment duration is based on clinical trials [4,25–27]			
	Alectinib (per 3 months)	€16,112	[28]
	Osimertinib (per 3 months)	€15,919	[28]
	Afatinib (per 3 months)	€5,883	[28]
	Lorlatinib (per 3 months)	€13,876	[28]

**End-of-life costs**			
	Per 3 months	€7,466	[29]
TKIs, tyrosine kinase inhibitors.			

Supplementary Table S8. Treatment utilization per lung cancer stage.

	Intervention	Utilization	Reference
**Stage I**	Surgery and/or chemotherapy	95%	[16,29]
	Chemo- and/or radiotherapy	5%	
**Stage II**	Surgery and/or chemotherapy	80%	[16,29]
	Chemo- and/or radiotherapy	20%	
**Stage III**	Surgery and/or chemotherapy	12%	[15,29,30]
	Chemo- and/or radiotherapy	54%	
	Chemo- and/or radiotherapy + durvalumab	20%	
	Immunotherapy	7%	[15,29,30]
	Immunochemotherapy	3%	
	Targeted therapy	4%	
**Stage IV**	Chemo- and/or radiotherapy	66%	[15,29,30]
	Immunotherapy	14%	
	Immunochemotherapy	10%	
	Targeted therapy	10%	

Supplementary Table S9. Follow-up costs for lung cancer patients.

**Item**	**Costs**
Chest CT scan [14]	€280
Pulmonologist consult [14]	€88
**Costs per cycle (3 months) in the follow-up years***	
Year 1-2 after initial treatments	184
Year 3-5 after initial treatments	92

CT, computed tomography.
* For Years 1-2 after initial treatments, patients received chest CT scans and pulmonologist consultation every 6 months, while for Years 3-5, patients received chest CT scans and pulmonologist consultation every 12 months, according to the clinical guideline for lung cancer treatments and surveillance [31].

Supplementary Table S10. Parameters and their corresponding values used for scenario analyses.

Corresponding parameters per scenario analysis		Value
Increase immunotherapy utilization by 10%		
	Stage III – utilization - Surgery and chemo - and/or radiotherapy	11.5%
	Stage III – utilization - Chemo- and/or radiotherapy	51.5%
	Stage III – utilization - Chemo- and/or radiotherapy + durvalumab	22.0%
	Stage III – utilization - Immunotherapy	7.7%
	Stage III – utilization - Immunochemotherapy	3.3%
	Stage III – utilization - Targeted therapy	4.0%
	Stage IV – utilization - Chemo- and/or radiotherapy	63.6%
	Stage IV – utilization - Immunotherapy	15.4%
	Stage IV – utilization - Immunochemotherapy	11.0%
	Stage IV – utilization - Targeted therapy	10.0%
	Hazard ratio overall survival stage III	0.80
	Hazard ratio overall survival stage IV	0.80
Increase immunotherapy utilization by 20%		
	Stage III – utilization - Surgery and chemo - and/or radiotherapy	10.9%
	Stage III – utilization - Chemo- and/or radiotherapy	49.1%
	Stage III – utilization - Chemo- and/or radiotherapy + durvalumab	24.0%
	Stage III – utilization - Immunotherapy	8.4%
	Stage III – utilization - Immunochemotherapy	3.6%
	Stage III – utilization - Targeted therapy	4.0%
	Stage IV – utilization - Chemo- and/or radiotherapy	61.2%
	Stage IV – utilization - Immunotherapy	16.8%
	Stage IV – utilization - Immunochemotherapy	12.0%
	Stage IV – utilization - Targeted therapy	10.0%
	Hazard ratio overall survival stage III	0.80
	Hazard ratio overall survival stage IV	0.80
Increase immunotherapy utilization by 50%		
	Stage III – utilization - Surgery and chemo - and/or radiotherapy	9.3%
	Stage III – utilization - Chemo- and/or radiotherapy	41.7%
	Stage III – utilization - Chemo- and/or radiotherapy + durvalumab	30.0%
	Stage III – utilization - Immunotherapy	10.5%
	Stage III – utilization - Immunochemotherapy	4.5%
	Stage III – utilization - Targeted therapy	4.0%
	Stage IV – utilization - Chemo- and/or radiotherapy	54.0%
	Stage IV – utilization - Immunotherapy	21.0%
	Stage IV – utilization - Immunochemotherapy	15.0%
	Stage IV – utilization - Targeted therapy	10.0%
	Hazard ratio overall survival stage III	0.80
	Hazard ratio overall survival stage IV	0.80
Increase immunotherapy utilization by 100%		
	Stage III – utilization - Surgery and chemo - and/or radiotherapy	6.5%
	Stage III – utilization - Chemo- and/or radiotherapy	29.5%
	Stage III – utilization - Chemo- and/or radiotherapy + durvalumab	40.0%
	Stage III – utilization - Immunotherapy	14.0%
	Stage III – utilization - Immunochemotherapy	6.0%
	Stage III – utilization - Targeted therapy	4.0%
	Stage IV – utilization - Chemo- and/or radiotherapy	42.0%
	Stage IV – utilization - Immunotherapy	28.0%
	Stage IV – utilization - Immunochemotherapy	20.0%
	Stage IV – utilization - Targeted therapy	10.0%
	Hazard ratio overall survival stage III	0.80

	Hazard ratio overall survival stage IV	0.80
Increase utility values for stage I lung cancer by 20%		
	Progression-free patients - utility value – stage I	0.94
	Progressed patients - utility value – stage I	0.83

References

1. Horeweg, N.; Van Der Aalst, C.M.; Vliegenthart, R.; Zhao, Y.; Xie, X.; Scholten, E.T.; Mali, W.; Thunnissen, E.; Weenink, C.; Groen, H.J.M.; et al. Volumetric Computed Tomography Screening for Lung Cancer: Three Rounds of the NELSON Trial. *European Respiratory Journal* **2013**, *42*, 1659–1667, doi:10.1183/09031936.00197712.

2. Yousaf-Khan, U.; Van Der Aalst, C.; De Jong, P.A.; Heuvelmans, M.; Scholten, E.; Lammers, J.W.; Van Ooijen, P.; Nackaerts, K.; Weenink, C.; Groen, H.; et al. Final Screening Round of the NELSON Lung Cancer Screening Trial: The Effect of a 2.5-Year Screening Interval. *Thorax* **2017**, *72*, 48–56, doi:10.1136/thoraxjnl-2016-208655.

3. Gandhi, L.; Rodríguez-Abreu, D.; Gadgeel, S.; Esteban, E.; Felip, E.; De Angelis, F.; Domine, M.; Clingan, P.; Hochmair, M.J.; Powell, S.F.; et al. Pembrolizumab plus Chemotherapy in Metastatic Non–Small-Cell Lung Cancer. *New England Journal of Medicine* **2018**, *378*, 2078–2092, doi:10.1056/NEJMOA1801005/SUPPL_FILE/NEJMOA1801005_DISCLOSURES.PDF.

4. Soria, J.-C.; Ohe, Y.; Vansteenkiste, J.; Reungwetwattana, T.; Chewaskulyong, B.; Lee, K.H.; Dechaphunkul, A.; Imamura, F.; Nogami, N.; Kurata, T.; et al. Osimertinib in Untreated EGFR -Mutated Advanced Non–Small-Cell Lung Cancer. *New England Journal of Medicine* **2018**, *378*, 113–125, doi:10.1056/nejmoa1713137.

5. Horn, L.; Mansfield, A.S.; Szczęsna, A.; Havel, L.; Krzakowski, M.; Hochmair, M.J.; Huemer, F.; Losonczy, G.; Johnson, M.L.; Nishio, M.; et al. First-Line Atezolizumab plus Chemotherapy in Extensive-Stage Small-Cell Lung Cancer. *New England Journal of Medicine* **2018**, *379*, 2220–2229, doi:10.1056/NEJMOA1809064/SUPPL_FILE/NEJMOA1809064_DATA-SHARING.PDF.

6. Goldstraw, P.; Chansky, K.; Crowley, J.; Rami-Porta, R.; Asamura, H.; Eberhardt, W.E.E.; Nicholson, A.G.; Groome, P.; Mitchell, A.; Bolejack, V.; et al. The IASLC Lung Cancer Staging Project: Proposals for Revision of the TNM Stage Groupings in the Forthcoming (Eighth) Edition of the TNM Classification for Lung Cancer. *J Thorac Oncol* **2016**, *11*, 39–51, doi:10.1016/j.jtho.2015.09.009.

7. McPherson, I.; Bradley, N.A.; Govindraj, R.; Kennedy, E.D.; Kirk, A.J.B.; Asif, M. The Progression of Non-Small Cell Lung Cancer from Diagnosis to Surgery. *European Journal of Surgical Oncology* **2020**, *46*, 1882–1887, doi:10.1016/j.ejso.2020.08.013.

8. Felip, E.; Altorki, N.; Zhou, C.; Csőszi, T.; Vynnychenko, I.; Goloborodko, O.; Luft, A.; Akopov, A.; Martinez-Marti, A.; Kenmotsu, H.; et al. Adjuvant Atezolizumab after Adjuvant Chemotherapy in Resected Stage IB–IIIA Non-Small-Cell Lung Cancer (IMpower010): A Randomised, Multicentre, Open-Label, Phase 3 Trial. *The Lancet* **2021**, *398*, 1344–1357, doi:10.1016/S0140-6736(21)02098-5.

9. Antonia, S.J.; Villegas, A.; Daniel, D.; Vicente, D.; Murakami, S.; Hui, R.; Yokoi, T.; Chiappori, A.; Lee, K.H.; de Wit, M.; et al. Durvalumab after Chemoradiotherapy in Stage III Non–Small-Cell Lung Cancer. *New England Journal of Medicine* **2017**, *377*, 1919–1929, doi:10.1056/nejmoa1709937.

10. Szende, A.; Janssen, B.; Cabasés, J. *Self-Reported Population Health: An International Perspective Based on EQ-5D*; Springer Netherlands, 2014; ISBN 9789400775961.

11. Marten, O.; Greiner, W. EQ-5D-5L Reference Values for the German General Elderly Population. *Health Qual Life Outcomes* **2021**, *19*, doi:10.1186/S12955-021-01719-7.

12. Burghuber, O.C.; Kirchbacher, K.; Mohn-Staudner, A.; Hochmair, M.; Breyer, M.K.; Studnicka, M.; Mueller, M.R.; Feurstein, P.; Schrott, A.; Lamprecht, B.; et al. Results of the Austrian National Lung Cancer Audit. *Clin Med Insights Oncol* **2020**, *14*, doi:10.1177/1179554920950548.

13. Kassenärztliche Bundesvereinigung Germany (National Association of Statutory Health Insurance Physicians). EBM. Available online: https://www.kbv.de/html/13259.php?srt=relevance&stp=fulltext&q=Bronchoskopie&s=Zoeken (accessed on 2 August 2023).

14. DHE Unit Cost Online Database: Cost Collection from Existing Studies. Version 3.1/2019. Vienna: Department of Health Economics (DHE), Center for Public Health, Medical University of Vienna.

15. Pirker, R.; Prosch, H.; Popper, H.; Klepetko, W.; Dieckmann, K.; Burghuber, O.C.; Klikovits, T.; Hoda, M.A.; Zöchbauer-Müller, S.; Filipits, M. Lung Cancer in Austria. *J Thorac Oncol* **2021**, *16*, 725–733, doi:10.1016/J.JTHO.2020.10.158.

16. Schwarzkopf, L.; Wacker, M.; Holle, R.; Leidl, R.; Günster, C.; Adler, J.B.; Huber, R.M. Cost-Components of Lung Cancer Care within the First Three Years after Initial Diagnosis in Context of Different Treatment Regimens. *Lung Cancer* **2015**, *90*, 274–280, doi:10.1016/J.LUNGCAN.2015.09.005.

17. Reck, M.; Rodríguez-Abreu, D.; Robinson, A.G.; Hui, R.; Csoszi, T.; Fülöp, A.; Gottfried, M.; Peled, N.; Tafreshi, A.; Cuffe, S.; et al. Updated Analysis of KEYNOTE-024: Pembrolizumab versus Platinum-Based Chemotherapy for Advanced Non–Small-Cell Lung Cancer with PD-L1 Tumor Proportion Score of 50% or Greater. *Journal of Clinical Oncology* **2019**, *37*, 537–546, doi:10.1200/JCO.18.00149.

18. Gadgeel, S.; Rodríguez-Abreu, D.; Speranza, G.; Esteban, E.; Felip, E.; Dómine, M.; Hui, R.; Hochmair, M.J.; Clingan, P.; Powell, S.F.; et al. Updated Analysis From KEYNOTE-189: Pembrolizumab or Placebo Plus Pemetrexed and Platinum for Previously Untreated Metastatic Nonsquamous Non-Small-Cell Lung Cancer. *J Clin Oncol* **2020**, *38*, 1505–1517, doi:10.1200/JCO.19.03136.

19. Antonia, S.J.; Villegas, A.; Daniel, D.; Vicente, D.; Murakami, S.; Hui, R.; Yokoi, T.; Chiappori, A.; Lee, K.H.; de Wit, M.; et al. Durvalumab after Chemoradiotherapy in Stage III Non–Small-Cell Lung Cancer. *New England Journal of Medicine* **2017**, *377*, 1919–1929, doi:10.1056/nejmoa1709937.

20. Socinski, M.A.; Nishio, M.; Jotte, R.M.; Cappuzzo, F.; Orlandi, F.; Stroyakovskiy, D.; Nogami, N.; Rodríguez-Abreu, D.; Moro-Sibilot, D.; Thomas, C.A.; et al. IMpower150 Final

Overall Survival Analyses for Atezolizumab Plus Bevacizumab and Chemotherapy in First-Line Metastatic Nonsquamous NSCLC. *J Thorac Oncol* **2021**, *16*, 1909–1924, doi:10.1016/j.jtho.2021.07.009.

21. Socinski, M.A.; Jotte, R.M.; Cappuzzo, F.; Orlandi, F.; Stroyakovskiy, D.; Nogami, N.; Rodríguez-Abreu, D.; Moro-Sibilot, D.; Thomas, C.A.; Barlesi, F.; et al. Atezolizumab for First-Line Treatment of Metastatic Nonsquamous NSCLC. *New England Journal of Medicine* **2018**, *378*, 2288–2301, doi:10.1056/NEJMOA1716948/SUPPL_FILE/NEJMOA1716948_DISCLOSURES.PDF.

22. McGahan, L. *Pembrolizumab (Keytruda®) as First-Line Therapy for PD-L1-Expressing, Locally Advanced or Metastatic Non-Small-Cell Lung Cancer (NSCLC). DSD: Horizon Scanning in Oncology 91.*; 2019;

23. Grössmann, N. *Atezolizumab (Tecentriq®) as Monotherapy for the First-Line Treatment of Adult Patients with Metastatic Non-Small Cell Lung Cancer (NSCLC). Oncology Fact Sheet Nr. 45.*; 2021;

24. McGahan, L. *Durvalumab (Imfinzi™) for the Treatment of Patients with Stage III Non-Small-Cell Lung Cancer after Prior Chemoradiotherapy*; 2017;

25. Mok, T.; Camidge, D.R.; Gadgeel, S.M.; Rosell, R.; Dziadziuszko, R.; Kim, D.-W.; Pérol, M.; Ou, S.-H.I.; Ahn, J.S.; Shaw, A.T.; et al. Updated Overall Survival and Final Progression-Free Survival Data for Patients with Treatment-Naive Advanced ALK-Positive Non-Small-Cell Lung Cancer in the ALEX Study. *Ann Oncol* **2020**, *31*, 1056–1064, doi:10.1016/j.annonc.2020.04.478.

26. Park, K.; Tan, E.H.; O'Byrne, K.; Zhang, L.; Boyer, M.; Mok, T.; Hirsh, V.; Yang, J.C.H.; Lee, K.H.; Lu, S.; et al. Afatinib versus Gefitinib as First-Line Treatment of Patients with EGFR Mutation-Positive Non-Small-Cell Lung Cancer (LUX-Lung 7): A Phase 2B, Open-Label, Randomised Controlled Trial. *Lancet Oncol* **2016**, *17*, 577–589, doi:10.1016/S1470-2045(16)30033-X.

27. Shaw, A.T.; Bauer, T.M.; de Marinis, F.; Felip, E.; Goto, Y.; Liu, G.; Mazieres, J.; Kim, D.-W.; Mok, T.; Polli, A.; et al. First-Line Lorlatinib or Crizotinib in Advanced ALK -Positive Lung Cancer. *New England Journal of Medicine* **2020**, *383*, 2018–2029, doi:10.1056/NEJMOA2027187/SUPPL_FILE/NEJMOA2027187_DATA-SHARING.PDF.

28. Österreichische Sozialversicherung (Austrian Social Insurance). Information Tool on the Reimbursement Code. Available online: https://www.sozialversicherung.at/oeko/views/index.xhtml (accessed on 1 August 2023).

29. Hofer, F.; Kauczor, H.U.; Stargardt, T. Cost-Utility Analysis of a Potential Lung Cancer Screening Program for a High-Risk Population in Germany: A Modelling Approach. *Lung Cancer* **2018**, *124*, 189–198, doi:10.1016/J.LUNGCAN.2018.07.036.

30. Wolf, A.; Stratmann, J.A.; Shaid, S.; Niklas, N.; Calleja, A.; Ubhi, H.; Munro, R.; Waldenberger, D.; Carroll, R.; Daumont, M.J.; et al. Evolution of Treatment Patterns and Survival Outcomes in Patients with Advanced Non-Small Cell Lung Cancer Treated at Frankfurt University Hospital in 2012-2018. *BMC Pulm Med* **2023**, *23*, doi:10.1186/S12890-022-02288-1.

31. Postmus, P.E.; Kerr, K.M.; Oudkerk, M.; Senan, S.; Waller, D.A.; Vansteenkiste, J.; Escriu, C.; Peters, S. Early and Locally Advanced Non-Small-Cell Lung Cancer (NSCLC): ESMO Clinical Practice Guidelines for Diagnosis, Treatment and Follow-Up. *Ann Oncol* **2017**, *28*, iv1–iv21, doi:10.1093/ANNONC/MDX222.
